# How I treat malignant pleural mesothelioma

**DOI:** 10.1136/esmoopen-2019-000669

**Published:** 2020-03-10

**Authors:** Giuseppe Viscardi, Raimondo Di Liello, Floriana Morgillo

**Affiliations:** Medical Oncology, Department of Precision Medicine, Università degli Studi della Campania Luigi Vanvitelli, Naples, Italy

**Keywords:** malignant pleural mesothelioma, mesothelioma

## Abstract

Malignant pleural mesothelioma is a rare and aggressive malignancy mostly associated with occupational asbestos exposure. Prognosis is poor and only highly selected patients may benefit from aggressive surgical management, also as part of a multimodal approach. In advanced disease, the combination of pemetrexed and platinum remains the only established treatment, while efficacy evidence of second line chemotherapy is lacking. Thus, a deeper knowledge of biology of the disease and more effective treatments are urgently needed. Refer to specialised centres with multidisciplinary expertise is mandatory, as well as inclusion of patients in clinical trials is advisable whenever possible. In all stages of disease focus on symptoms control is paramount.

## Introduction

Malignant pleural mesothelioma (MPM) is a relatively rare tumour strictly correlated to occupational exposure to asbestos that accounts for almost 80% of the cases. Its incidence has risen steadily in the last years mainly due to the latency time estimated up to 50 years after exposure. Although processing of asbestos has been banned at least in many western countries, a peak is predicted in the next decade.^1^ More recently, somatic or germline mutations of *BAP1* (BRCA1-associated protein 1) gene have been described as predisposing factor for MPM.^2^ Prognosis remains poor, with a survival rate at 5 years <10%.

Histologically heterogeneous, most MPMs (50%–60%) are represented by the epithelial subtype, whereas approximately 10% are sarcomatoid and the remainder biphasic, with the sarcomatoid histology having the worst survival.

Non-spherical shape of tumour and unconventional pattern of growth makes current TNM staging system difficult to apply to clinical staging. Also assessment of response to treatments is challenging, and modified RECIST criteria (Response Evaluation Criteria in Solid Tumors) adapted to MPM have been proposed.

**Figure 1:**
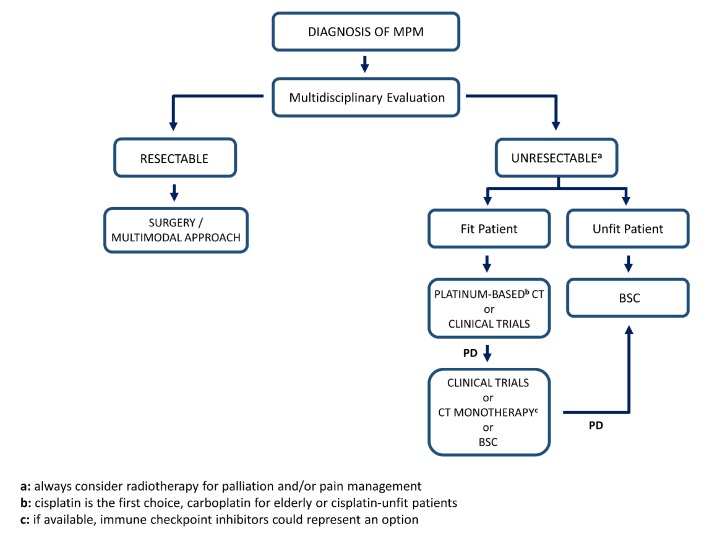
Proposed algorithm for management of MPM. MPM, malignant pleural mesothelioma; PD, progressive disease; CT, chemotherapy; BSC, best supportive care.

Patients with pathologically confirmed diagnosis of MPM should be referred to specialised centres with multidisciplinary expertise and high volume of cases (figure 1).

## Resectable disease

Macroscopic complete resection (MCR), possibly given as part of multimodal approach, remains one of the options in mesothelioma treatment, although its real benefit is still a matter of debate as no randomised study to date has shown a survival advantage in patients undergoing surgery.

MCR can be achieved by extrapleural pneumonectomy (EPP) or lung sparing pleurectomy with decortication (P/D), eventually extended to removal of diaphragm and pericardium.^3^

The ideal candidate to resection with curative intent has good performance status (PS), compatible cardio-pulmonary reserve, pure epithelial histology and low tumour burden with absence of lymph node involvement.

In a systematic review of the use of EPP the median overall survival (mOS) varied from 9.4 to 27.5 months, and 5-year survival rates from 0% to 24%; overall mortality ranged from 0% to 11.8% and morbidity from 22% to 82%.^4^

A shift towards P/D as surgical modality for MPM has been observed in the last decade. Comparison of P/D to EPP remains challenging due to the absence of randomised trials, but P/D seems to have less mortality and morbidity, with comparable overall and disease-free survival.^5^

Some studies reported a trimodality approach, including neoadjuvant chemotherapy, EPP and postoperative radiation therapy (RT), either as haemithorax radiation or as intensity modulated RT (IMRT). In a systematic review encompassing 16 studies, the median OS ranged from 12.8 to 46.9 months with perioperative mortality from 0% to 12.5%.^6^ Preoperative chemotherapy could increase the complete resection rate of early-stage mesothelioma and radiotherapy exert an addictive effect. Due to surgery morbidity, patient selection for completion of trimodal treatment represents a critical factor. IMRT after EPP is promising as good local control can be obtained and organs at risk well protected; moreover, IMRT to the entire pleura seems to be feasible after P/D.

However, the results of the multicentre randomised Mesothelioma and Radical Surgery 1 (MARS 1) trial failed to provide evidence of benefit for survival or quality of life from EPP within trimodal therapy over chemotherapy alone,^7^ whereas the MARS 2 trial is currently assessing the role of P/D in the context of a multimodal approach.

## Unresectable disease

Management of unresectable MPM includes both systemic and local therapy although few treatment options are available.

Front-line polichemotherapy is considered the standard of care, whereas single-agent chemotherapy has shown limited efficacy with disappointing response rates. A pivotal trial by Vogelzang *et al* led to the establishment of cisplatin and pemetrexed as standard first line regimen for unresectable MPM. Median progression-free survival (mPFS) and mOS were significantly longer in pemetrexed/cisplatin arm versus cisplatin alone (5.7 vs 3.9 months, p=0.001 and 12.1 vs 9.3 months, p=0.020, respectively), as well as response rate (41.3% vs 16.7%, p<0.0001).^8^

Carboplatin appears a reasonable alternative to cisplatin in elderly or unfit population, exhibiting comparable response and survival rates when combined with pemetrexed.^9^ The association of gemcitabine and cisplatin has also been investigated in phase II trials and may be a reasonable option for patients who are unable to tolerate pemetrexed.^10^

Unlike non-squamous non-small-cell lung cancer, the role of maintenance treatment with antifolates remains unclear for MPM patients. Despite the so-called ‘Rotterdam experience’ documented the feasibility and good tolerability of this strategy, a recent phase II trial from Cancer and Leukemia Group B (CALGB) showed that pemetrexed continuation after 4–6 cycles of doublet chemotherapy induction did not prolong PFS over placebo (mPFS 3.4 vs 3.0 months, p=0.9733).^11^ Although positive results came from a phase II trial (NVALT19) assessing the role of gemcitabine switch maintenance with a PFS benefit of 2.5 months (HR 0.42, p<0.0001),^12^ overall, these data do not support at the moment the use of any maintenance therapy for unresectable MPM after induction chemotherapy.

Angiogenesis also has been extensively investigated in MPM. Adding bevacizumab to first line platinum doublet resulted in a modest PFS and OS gain in the phase III French trial MAPS with a concomitant increase of drug-related toxicity.^13^ Results of phase III LUME-Meso study failed to confirm data from the phase II part, since addition of nintedanib to standard front-line chemotherapy did not prolong PFS in patients with epithelioid MPM.^14^

Focus on maintaining quality of life and pain control is paramount. Patients with MPM have a relevant symptom burden (fatigue, dyspnoea, pain, cough, anorexia) requiring opioid analgesia and an earlier integration of palliative and supportive care at all disease stages.

A persistent pleural fluid effusion may be managed by performing a talc pleurodesis (via chest tube or thoracoscopy) which is highly efficient when lung re-expansion is obtained. Pleurodesis is also associated with fewer complications compared with video assisted thoracic surgery pleurectomy.^15^ Placement of indwelling pleural catheter is a suitable option for trapped lung syndrome.

Radiotherapy should be considered in all patients with localised disease causing pain or obstructive symptoms, usually with hypofractionated regimens.^16^

Appearance of painful subcutaneous tumour nodules may be the consequence of malignant cells seeding along instrument tracts at sites of diagnostic or therapeutic intervention. In the largest trial investigating the use of prophylactic radiotherapy to prevent procedure-tract metastases (SMART trial), no benefits in terms of symptom control and survival was observed compared with deferred radiotherapy in overall population, thus the use of routine prophylactic radiotherapy for all patients is not recommended.^17^

### Second line systemic therapy

Unfortunately, nearly all patients progress during or after first-line therapy, and no standard second line recommended treatments exist after platinum-based regimen.

Retreatment with pemetrexed, eventually associated with platinum-compound, may be offered in patients who achieved durable disease control (>6 months) with first line chemotherapy.^18^

Single-agent chemotherapy with vinorelbine or gemcitabine is the preferred choice of most of physicians mainly based on retrospective analyses or small phase II trials with a response rate of 15%–20%, median PFS of about 2 months and median of OS 6–9 months.^19^

Thus, evaluation for enrolment in clinical trials represents an advisable option for patients relapsing after first-line treatment, whereas patients with an Eastern Cooperative Oncology Group (ECOG) PS of three or greater should receive palliative care only.

## Immunotherapy

While immune checkpoint inhibitors (ICIs) represent a standard therapeutic modality in many other solid tumours, outcomes in MPM have been less positive and may be influenced by the complex structure of tumour microenvironment.

In the salvage setting encouraging results came from single-arm clinical trials targeting programmed cell death 1 (PD-1) or programmed death-ligand 1 (PD-L1), showing response rates ranging from 9.4% to 29.4% and a small proportion of long-term responders. However, in randomised clinical trials the cytotoxic T-lymphocyte antigen 4 (CTLA-4) inhibitor tremelimumab^20^ and the PD-1 antibody pembrolizumab^21^ failed to improve PFS in pretreated patients, respectively over placebo and standard chemotherapy.

Moving forward from single agent checkpoint blockade, ongoing combination strategies include combination of PD-1/PD-L1 antibodies with chemotherapy, anti CTLA-4, or targeted therapy such as FAK (Focal Adhesion Kinase) or AXL inhibitors and antiangiogenic drugs.

The combination of durvalumab, cisplatin and pemetrexed as first line of treatment in the single-arm phase II DREAM trial has demonstrated sufficient activity, exhibiting mPFS of 6.9 months and median duration of response of 6.5 months and safety, with no deaths attributed to durvalumab and neutropenia, nausea and anaemia presenting as more commons grade ≥3 adverse events.^22^ So, this strategy is currently under investigation in the randomised phase III trial DREAM3R, whereas the phase III trial led by Canadian Cancer Trials Group IND.227 is evaluating the efficacy of upfront pembrolizumab plus chemotherapy.

The addition of anti CTLA-4 to anti-PD-1/PD-L1 seems to add a modest increment in overall response rate, with only one randomised study (MAPS-2) reporting a signal for longer mPFS (5.6 vs 4.0 months) and mOS (15.9 vs 11.9 months) in nivolumab–ipilimumab arm compared with nivolumab alone in patients with relapsed MPM.^23^

No predictive biomarkers of response have been defined for ICIs.

## Perspectives

In the next years growth in understanding of mesothelioma biology is expected to lead therapeutic developments (table 1).

**Table 1:** New strategies currently under investigation in malignant pleural mesothelioma treatment

**Systemic treatments**	
Strategy under investigation	Biomarker
Arginine deiminase	ASS1 deficiency
EZH2, PARP or HDAC inhibitors	BAP-1 mutations
CDK4/6 inhibitors	CDKN2A mutations
Mesothelin-targeted therapy	Mesothelin overexpression
FAK inhibitors	NF-2 mutations
PI3K/mTOR inhibitors	PI3K/AKT/mTOR pathway activation
Immune checkpoint inhibitors (single-agent or combinations)	Not established
Adoptive immunotherapy	Overexpressed differentiation antigens
New chemotherapy drugs (trabectedin, lurbinectedin)	No druggable alterations
**Loco-regional treatments**	
Tumour treatment fields (TTF)	
Intracavitary therapies	
Neoadjuvant radiation therapy.	

ASS1, Argininosuccinate Synthase 1; BAP1, BRCA 1-Associated Protein 1; CDK4/6, Cyclin-Dependent Kinase 4/6; CDKN2A, Cyclin-Dependent Kinase inhibitor 2A; EZH2, Enhancer of Zeste Homolog 2; HDAC, Histone DeACetylases; mTOR, mammalian Target Of Rapamycin; NF-2, NeuroFibromin-2; PARP, Poly ADP-Ribose Polymerase; PI3K, PhosphoInositide 3-Kinase.

MPM is characterised by a low mutational burden, as detected by standard sequencing approaches, and a tumour microenvironment rich of immunosuppressive cells and anergic signals. Combining ICIs with other agents may help to overcome these barriers and improve their limited clinical response.

Furthermore, the genomic landscape is dominated by inactivation of several tumour suppressors genes. In particular, mutations of *CDKN2A*, *BAP1* and *NF2* are three of the most frequent genomic alterations detected, for which novel drugs are under investigation.

Research based on tumour metabolism is focusing on arginine deprivation in argininosuccinate synthase 1 (ASS1) deficient tumours.

Finally, adoptive T cell therapy is another promising cell-based strategy. Overexpressed differentiation antigens, such as mesothelin, or components of the tumour-associated stroma, such as fibroblasts or endothelial cells, represent attractive targets for chimeric antigen receptor T cell therapy.
